# How to Build a Hybrid Neurofeedback Platform Combining EEG and fMRI

**DOI:** 10.3389/fnins.2017.00140

**Published:** 2017-03-21

**Authors:** Marsel Mano, Anatole Lécuyer, Elise Bannier, Lorraine Perronnet, Saman Noorzadeh, Christian Barillot

**Affiliations:** ^1^Institut National de Recherche en Informatique et en Automatique (INRIA)Rennes, France; ^2^Institut de Recherche en Informatique et Systèmes Aléatoires (IIRISA)Rennes, France; ^3^CHU PontchaillouRennes, France; ^4^Institut National de la Santé et de la Recherche MédicaleRennes, France

**Keywords:** neurofeedback, electroencephalography, functional magnetic resonance imaging, multimodal, brain signals, real-time

## Abstract

Multimodal neurofeedback estimates brain activity using information acquired with more than one neurosignal measurement technology. In this paper we describe how to set up and use a hybrid platform based on simultaneous electroencephalography (EEG) and functional magnetic resonance imaging (fMRI), then we illustrate how to use it for conducting bimodal neurofeedback experiments. The paper is intended for those willing to build a multimodal neurofeedback system, to guide them through the different steps of the design, setup, and experimental applications, and help them choose a suitable hardware and software configuration. Furthermore, it reports practical information from bimodal neurofeedback experiments conducted in our lab. The platform presented here has a modular parallel processing architecture that promotes real-time signal processing performance and simple future addition and/or replacement of processing modules. Various unimodal and bimodal neurofeedback experiments conducted in our lab showed high performance and accuracy. Currently, the platform is able to provide neurofeedback based on electroencephalography and functional magnetic resonance imaging, but the architecture and the working principles described here are valid for any other combination of two or more real-time brain activity measurement technologies.

## 1. Introduction

Neurofeedback (NFB), also called neuro-therapy or neurobiofeedback, is defined as the self-regulated change of a particular brain activity that is reflected in the change of one or several neurosignals captured by brain activity measurement technologies such as electroencephalography (EEG), blood-oxygen-level dependent (BOLD) functional magnetic resonance imaging (fMRI), magnetoencephalography (MEG), or near-infrared spectroscopy (NIRS). Taking advantage of the human brain plasticity, the aim of NFB is to treat or cure neurological and neuropsychiatric disorders with learned self-regulation of the disordered brain regions. Commonly provided visually or aurally, NFB achieves its goal by presenting the subject with his/her estimated brain activity in real-time (Strehl et al., 2006; Birbaumer et al., 2013; Stoeckel et al., 2014). Various NFB studies have been reported in both research and clinical domain (Evans and Abarbanel, 1999; Kotchoubey et al., 2001; Christopher deCharms et al., 2005; Strehl et al., 2006; Haller et al., 2010; Subramanian et al., 2011; Linden et al., 2012; Sitaram et al., 2012; Weiskopf, 2012; Li et al., 2013; Ruiz et al., 2013).

The majority of the existing studies employ only one measurement technology to estimate the NFB. Such unimodal NFB focusses on a single aspect of the neurophysiological processes, such as brain electrophysiological activity for EEG or hemodynamic activity for fMRI.

Recently, there has been a growing interest in the NFB community to combine more than one brain activity measurement technology (Nakano et al., 2012; Yu et al., 2013; Jorge et al., 2014; Koo et al., 2015), where the combination of EEG and fMRI seems to be the most notable. For instance, fMRI has been used as the second complementary data in the EEG inverse problem (Grech et al., 2008) to localize the origins of the neuronal activity. Since the inverse problem is undetermined (Vogel, 2002), fMRI has been used as a constraint to obtain a solution to the problem. The combinational approaches can be fMRI-constrained in which fMRI derived spatial priors are used in EEG source identification (Bießmann et al., 2011), or they can be the simultaneous fusion of the modalities (Valdes-Sosa et al., 2009). Furthermore, new mathematical and statistical approaches that use multimodal EEG-fMRI fusion and benefit from their complementary natures, are being developed. Such methods include joint independent component analysis (ICA) (Calhoun et al., 2006) or ICA based group inferences from fMRI (Calhoun et al., 2009). In another recent approach, a general framework for the fusion of EEG and fMRI, is introduced (Karahan et al., 2015). These multimodal observations that capture different aspects of the neurophysiological activity have the potential to provide additional information about the ongoing brain activity, consequently leading to better NFB estimation. For instance, in the case of EEG-fMRI, while the EEG measurements offer good temporal but poor spatial information, the fMRI measurements offer good spatial but little temporal information. Combining the strongest aspects of each modality can potentially provide a better estimate than any of them separately. The majority of the existing bimodal EEG-fMRI studies estimate the real-time NFB only from one modality (usually EEG) while still using both modality measurements for offline analysis (Kinreich et al., 2012; Ros et al., 2013; Meir-Hasson et al., 2014; Shtark et al., 2015; Zich et al., 2015; Keynan et al., 2016; Zotev et al., 2016). The only study that estimates bimodal NFB in real-time is reported by Zotev et al. (2014). To the best of our knowledge there is no commercial system that offers bimodal EEG-fMRI NFB.

In this paper we describe how to set up and use a bimodal NFB platform. Furthermore, we provide a general overview of the main technical challenges and explain how we have addressed them during the development of our bimodal EEG and fMRI platform at Neurinfo^1^.

## 2. General description of a hybrid EEG-fMRI platform for bimodal neurofeedback

**Figure 1:**
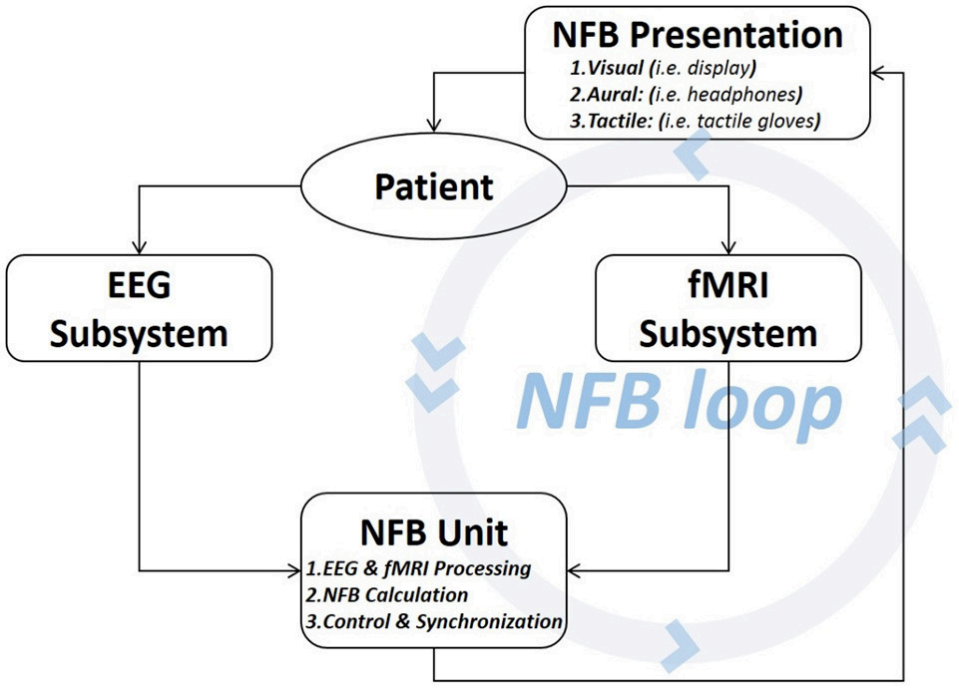
**Abstract diagram of an hybrid EEG-fMRI NFB platform**. The patient/subject is an integral part of the NFB loop. The brain activity generates neurosignals which are read with two subsystems then forwarded to the NFB Unit. This unit estimates the NFB and then shows the results to the patient through the display, thus allowing self-regulation of the brain activity based on the real-time NFB.

The abstract diagram of a hybrid EEG-fMRI NFB platform is shown in Figure 1. A hybrid platform must have a magnetic resonance (MR) compatible EEG and an fMRI acquisition subsystems. Such subsystems are commercially available (see Sections 3.1 and 3.2) and must be acquired with all the necessary components that enable real-time acquisition.

The platform must also have an *NFB Unit* that is capable of: (1) connecting and acquiring brain signals coming from each subsystem in real-time, (2) estimating the NFB values from the brain signals, (3) handling the configuration and execution of the experimental protocol, (4) ensuring full synchronization, and (5) establishing continuous communication with the subject. The *NFB Unit* is rather a logical unit that can be implemented by using different software modules deployed on one or several computers/servers on a network.

The NFB loop is closed with the communication device (i.e., display or headphones) which presents the NFB to the patient.

### 2.1. *NFB Unit*

The *NFB Unit* should provide for each modality a real-time processing pipeline that handles signal acquisition and all the necessary methods/algorithms required for NFB calculation. Furthermore, it should provide the flexibility of using multimodal or unimodal NFB. The following section explains in detail the *NFB Unit* components and their functions.

#### 2.1.1. The real-time EEG processing pipeline

**Figure 2:**
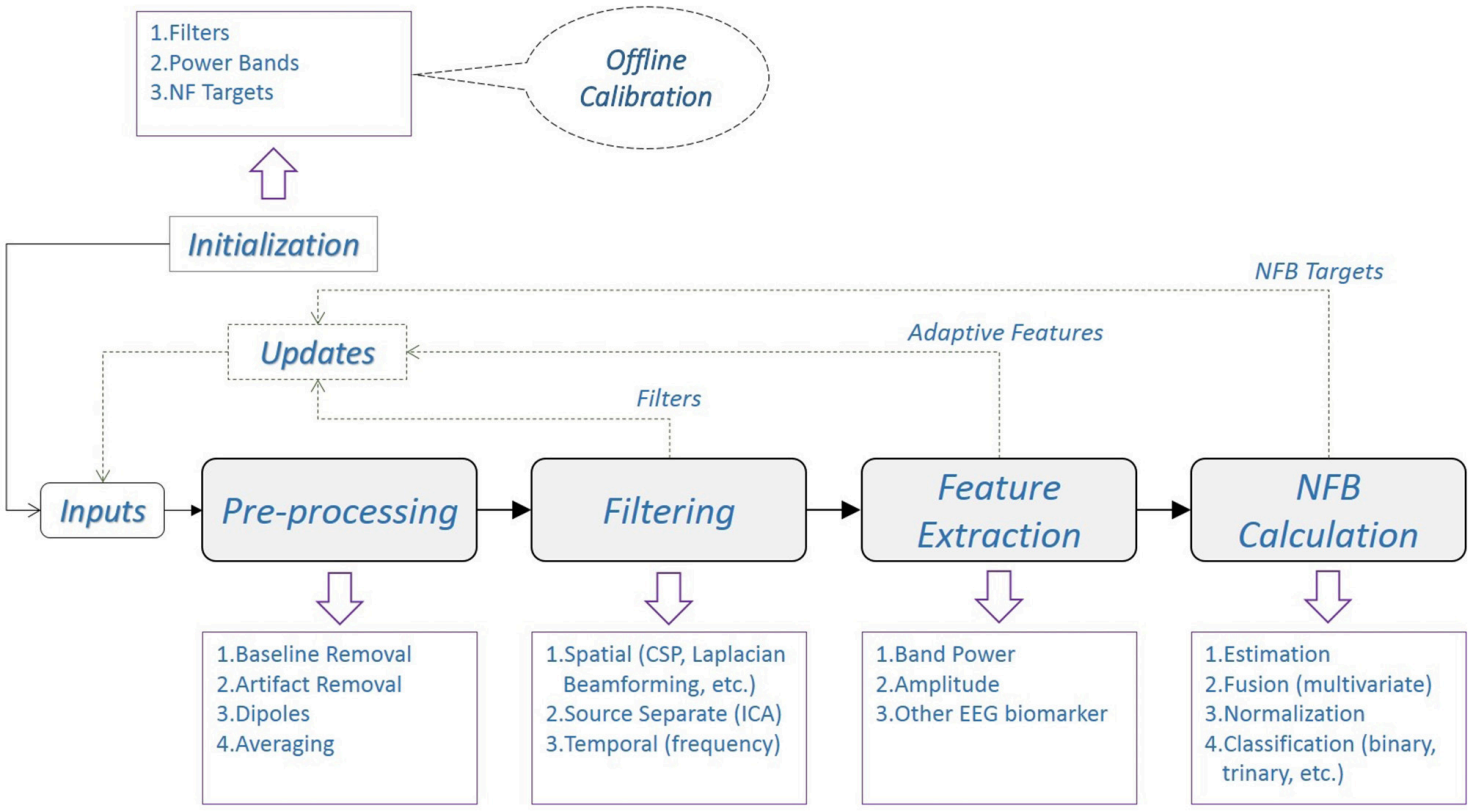
**Generic diagram of a real-time EEG processing pipeline for NFB**. The (optional) offline calibration, usually performed right before the real-time experiments, is used to obtain initialization information for real-time processing during NFB sessions. At each stage of the pipeline there are few examples of signal processing methods, shown inside the purple boxes. The most common types of (optional) updates from each stage are also indicated with dashed lines.

The exact pipeline architecture and its implementation heavily depend on (i) the NFB application(s), (ii) the selected EEG subsystem and (iii) the individual software engineering approach. A generic diagram of the real-time EEG processing pipeline for NFB is shown in Figure 2. This diagram is not a rigid design architecture but rather a guideline for the real-time EEG processing flow. Furthermore, its components can be implemented as separate software modules and/or deployed on several processing hardware. The pipeline architecture promotes parallel computing of different signal processing steps, which improves real-time performance.

##### 2.1.1.1. Initialization

The initialization information is usually obtained through a preliminary offline training or calibration session (see Section 4) but it can also include a priori information based on empirical knowledge about EEG and/or NFB. Depending on the experimental protocol and the selected signal processing techniques, the initialization might include spatial or temporal filters, band power estimates, signal components (i.e., principal or independent components), thresholds, features or NFB targets.

##### 2.1.1.2. Updates

Some NFB protocols require online updates that complement or even substitute the initialization information throughout the duration of an experiment. These updates can be used to improve the EEG signal filtering, reevaluate the extracted features or change the NFB targets.

##### 2.1.1.3. Pre-processing

This step includes various preliminary signal processing operations (i.e., artifact and/or baseline removal, dipole extraction, etc.) that aim at improving the signal-to-noise ratio in EEG by minimizing the effect of local and/or global artifacts. Indeed, there are some distinctive EEG artifacts that occur only during simultaneous EEG and fMRI acquisition, which can severely compromise the quality of the EEG signals.

- The *gradient artifacts* are caused by the scanner's alternating gradient magnetic field during an MR acquisition. The very high amplitude and frequency variability range of the gradient magnetic field causes artifacts with amplitude often more than 100 times higher than normal EEG. During an fMRI acquisition the gradient artifact pattern within each Time of Repetition (TR) is ideally identical, which leads to very low inter-volume variability generated gradient artifacts. Hence, the EEG signals recorded within a TR window can be filtered by subtracting the artifact as a template (Allen et al., 2000). The template is estimated by averaging a user defined number consecutive equally spaced intervals extracted in phase with the artifact generation. This method causes the randomly distributed EEG signals to be subtracted from the averaged curve, ideally leaving only the external influence of the scanner. For online applications, 5–15 shifting consecutive TRs are used to build the template. The template is then subtracted from the following TR window, thus leaving only the filtered EEG signal. It is worth noting that the EEG amplifiers should be very sensitive to small changes in EEG micro currents (~0.1μV), while also having a large dynamic amplitude range (~50,000 μV) in order to record both the EEG with appropriate resolution and the MR artifacts without saturation.
- The *ballistocardiogram (BCG) artifacts* are caused by the micro currents generated by the pulsatile blood flow related movement of the EEG electrodes in the presence of the strong magnetic field of the scanner. Their occurrence is thus strongly related to the subject's heartbeat and their amplitude range is higher than that of normal EEG. The BCG artifacts correction method is very similar to that of gradient artifact. Heartbeats are recorded and detected on a specific channel, then for each channel a template is calculated using 10–20 pulse intervals. Finally, the template is removed from the following pulse interval (Allen et al., 1998). Depending on the subject's heartbeat variability, the removal of the BCG artifacts can be very challenging and give less than optimal results during real-time applications.
- Another MR specific EEG artifact is caused by the scanner's internal ventilation system. The best way to avoid this artifact is to switch off the ventilation system for the duration the experiment, if this is allowed by the MR scanner manufacturer's guidelines. Otherwise, it can be removed as shown by Nierhaus et al. (2013).
- Last, any other type of voluntary or involuntary head motion of the patient inside the MR scanner can cause irregular EEG artifacts that can be very hard to detect and remove. There exist some practical methods that remove the motion artifacts not only in EEG signals (Nakamura et al., 2006; Jorge et al., 2015; Klovatch-Podlipsky et al., 2016), but also in fMRI by using the motion estimation extracted from EEG signals (Zotev et al., 2012).

After removing all the MR specific artifacts, the real-time EEG signal processing is very similar to that of the standard EEG acquired outside of MR.

##### 2.1.1.4. Filtering

This step includes more elaborate signal processing operations in the spatial and temporal domain. Commonly used spatial filtering techniques include variants of surface Laplacian, common spatial patterns (CSP), or beamforming (Spencer et al., 1992; Nunez and Westdorp, 1994; Lotte and Guan, 2011). More elaborate techniques that aim at EEG source localization and signal decomposition use various independent component analysis (ICA) methods, inverse modeling, etc. (Pascual-Marqui et al., 2002; Subasi and Gursoy, 2010). Temporal filtering is usually based on the spectral analysis of the EEG signals. The majority of the filtering operations requires preliminary training to build subject specific filters and/or mathematical models in order to improve the real-time filtering results.

##### 2.1.1.5. Feature extraction

After filtering, predefined EEG features are extracted. The choice of features highly depends on the NFB protocol. The most common features are extracted from the signal power analysis in the frequency domain. The features are then used for the NFB calculation (see Section 2.1.3).

#### 2.1.2. The real-time fMRI processing pipeline

**Figure 3:**
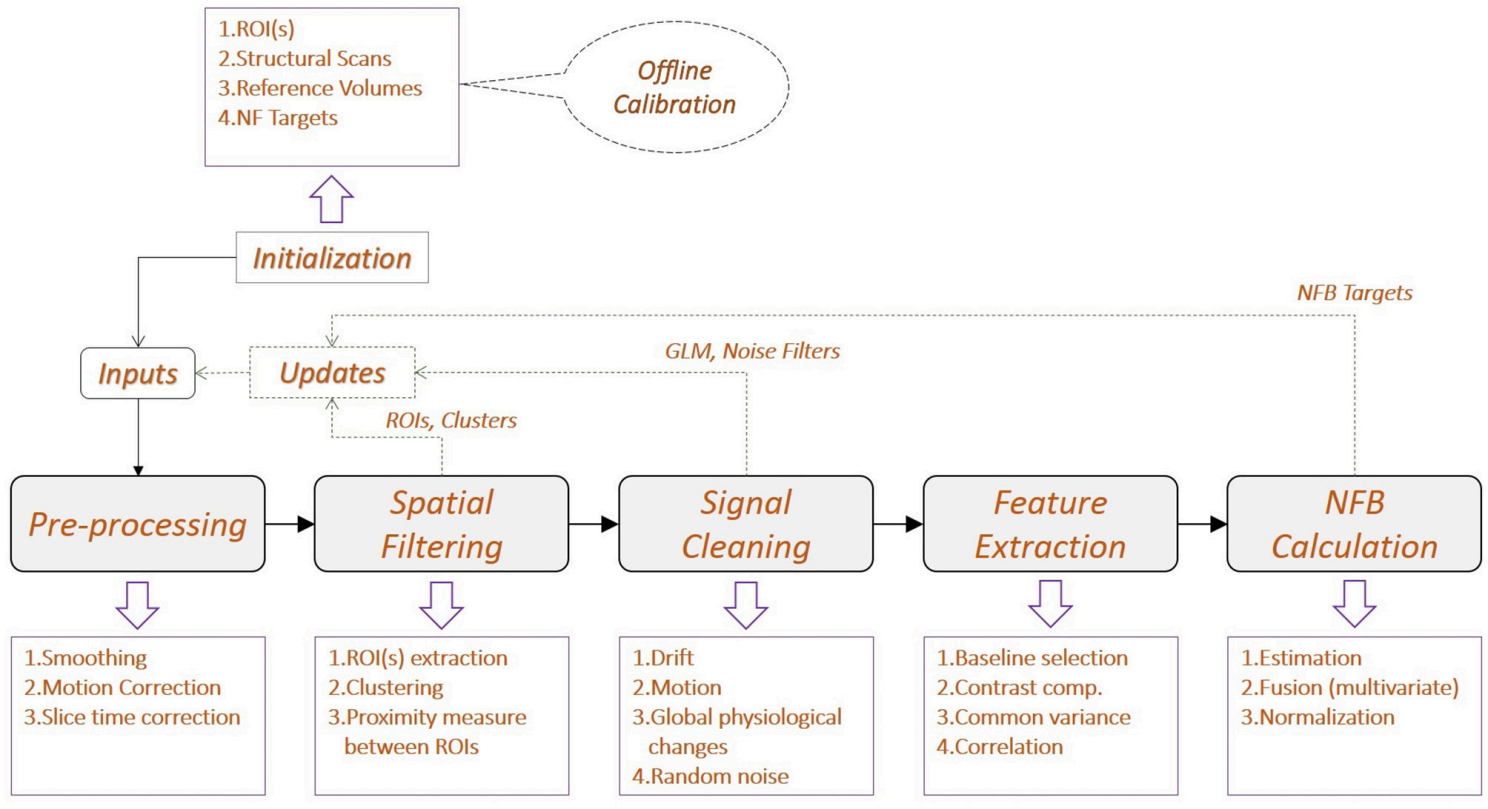
**Generic diagram of a real-time fMRI processing pipeline for NFB**. The same principles shown for EEG in Figure 2 apply also here for fMRI. Furthermore, depending on the fMRI processing method, the spatial and temporal filtering are sometimes interchangeable.

The diagram of a generic real-time fMRI processing pipeline for NFB is shown in Figure 3. Similar to EEG, the fMRI pipeline architecture also depends on the application, the fMRI subsystem and software engineering approach.

##### 2.1.2.1. Initialization

The fMRI initialization information is spatial, temporal or a combination of the two. A typical spatial information is a brain region of interest (ROI) that can be selected a priori (i.e., from a brain atlas, Cox, 1996; Tzourio-Mazoyer et al., 2002), extracted offline from previous studies or by a functional localizer preceding the NFB session. Examples of temporal information are the experimental design, the hemodynamic response function as well as various temporal filters used for time-series analysis. The fMRI activation mapping techniques yield both spatial and temporal information. There are also some protocols that would take into account different ROIs and their activation order based on dynamic causal models (Penny et al., 2004; Stephan et al., 2008; Penny et al., 2011). Initialization may also include information used for NFB estimation like target BOLD contrast values or thresholds.

##### 2.1.2.2. Updates

Real-time updates can be used to improve spatial filtering by using voxel clustering in neighboring areas or ROI shape and size changes, to improve temporal filtering (i.e., change online processing parameters or noise filters), or even to dynamically change the NFB target(s).

##### 2.1.2.3. Pre-processing

This step includes various mathematical transformations of the fMRI volume series including registration, motion estimation and correction, smoothing and slice-time correction. Their aim is to improve the fMRI signal-to-noise ratio and also to account for signal distortion due to subject's head motion (Friston et al., 1995; Jenkinson et al., 2002).

##### 2.1.2.4. Spatial filtering

Global brain activity is seldom the goal of NFB. Instead, local activities on specific ROI(s) are usually monitored. Spatial filtering is used to extract the BOLD contrast values of the ROI voxels. This provides focus to the hemodynamic activity of the targeted brain region(s). Furthermore, it also reduces significantly the online computation demand for the following processing steps.

##### 2.1.2.5. Temporal filtering

The fMRI signal is affected by random noise, physical artifacts from the scanner, subject's motion artifacts or other physiological fluctuations (Bianciardi et al., 2009). The random noise can be removed by using Gaussian smoothing or temporal averaging; the scanner drift by linear trend removal, exponential moving average (Roberts, 2000; Cui et al., 2010; Koush et al., 2012), high-pass filtering, correlation analysis or generalized linear model (GLM) analysis. Global and local physiological fluctuations can also be removed by subtracting background activity, temporal filtering, or again GLM analysis with confound predictors. Standard offline SPM^2^ processing uses GLM analysis to linearly fit the whole fMRI time series into a set of specific time-series components pre-defined in the design matrix, followed by an activation mapping process based on statistical and spatial analysis of the GLM results. In real-time experiments, similar modeling like online windowed GLM (Nakai et al., 2006) or the incremental GLM (Bagarinao et al., 2003), can be done by using the acquired signal instead of the whole fMRI time series. These methods perform a new GLM based analysis for each new fMRI volume. Other online fMRI methods use correlation analysis (Cox et al., 1995; Gembris et al., 2000) or ICA (Esposito et al., 2003; Chiew, 2013; Soldati et al., 2013a,b).

##### 2.1.2.6. Feature extraction

Predefined features that will be used for NFB estimation, are extracted from the filtered signal. The features, their extraction and how they are used for the NFB estimation is determined by the experimental protocol. Commonly, these are statistical observations or inferences over the ROI(s), i.e., the maximum likelihood, z-score or *p*-values. Some protocols use more elaborated spatial analysis based features like sub-clustering.

#### 2.1.3. NFB calculation

**Figure 4:**
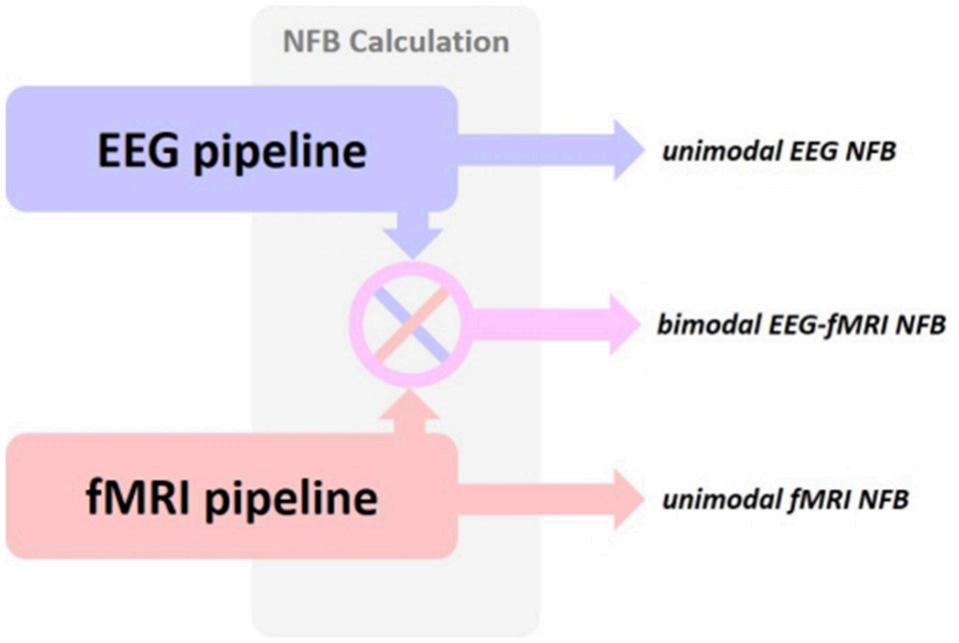
**The hybrid platform should able to provide both unimodal and bimodal NFB based on the requirements of the NFB protocol**. Depending on the protocol, the output can be: i) unimodal EEG NFB, ii) unimodal fMRI NFB, or iii) bimodal EEG-fMRI NFB. Switching between them could be done with a simple initial configuration.

The bimodal NFB platform must be able to provide also unimodal EEG or fMRI NFB by using only one modality (see Figure 4). In this section we will consider some of the most commonly used NFB targets for both EEG and fMRI and how to estimate the NFB values accordingly.

The NFB target or goal is strictly related to the experimental or clinical protocol, hence they can be quite different and used in a variety of applications (see Section 1). Furthermore, they can be either constant or dynamically changed throughout the duration of the experimental session.

Generally, the NFB value is estimated using the features extracted from their respective pipelines (see Figures 2, 3). In unimodal NFB, only one set of features is considered, and NFB can be estimated as a relative or normalized measure between the current value and the target [i.e., average percent signal change (APSC); Christopher deCharms et al., 2005; Hinds et al., 2011], a normalized statistic observations or a connectivity measure (Koush et al., 2013; Zilverstand et al., 2014). In the case of unimodal EEG-NFB protocols that aim at the increase (decrease) of a certain neural oscillation at a specific frequency band and at a specific measuring or source location, the NFB value can be the estimation of the relative change of the EEG spectral power related features. Similarly, for unimodal fMRI-NFB protocols that aim the increase (decrease) of the hemodynamic activity at a specific ROI, the NFB value can be estimated as the relative BOLD contrast change of the ROI voxels. Other fMRI-NFB protocols aim to reach a certain signal change at specific brain regions or even spatial augmentation of these regions, in such cases the NFB value can be estimated as the relative change of the ROI size (Yoo and Jolesz, 2002). More elaborate protocols rely on the functional connectivity of various ROIs or causal modeling (Koush et al., 2013).

In bimodal protocols, the calculation can be done separately for each modality and then the two NFB values are: (1) given as a two dimensional vector [EEG-NFB, fMRI-NFB], or (2) combined together mathematically to give a one dimensional NFB. Another possibility is to combine the features of each modality and use them as the input of a joint model that estimates unidimensional NFB. There also exists the possibility to use a joint EEG-fMRI modeling approach to extract features from both EEG and fMRI signals simultaneously and then estimate NFB based on the joint features.

#### 2.1.4. Synchronization

**Figure 5:**
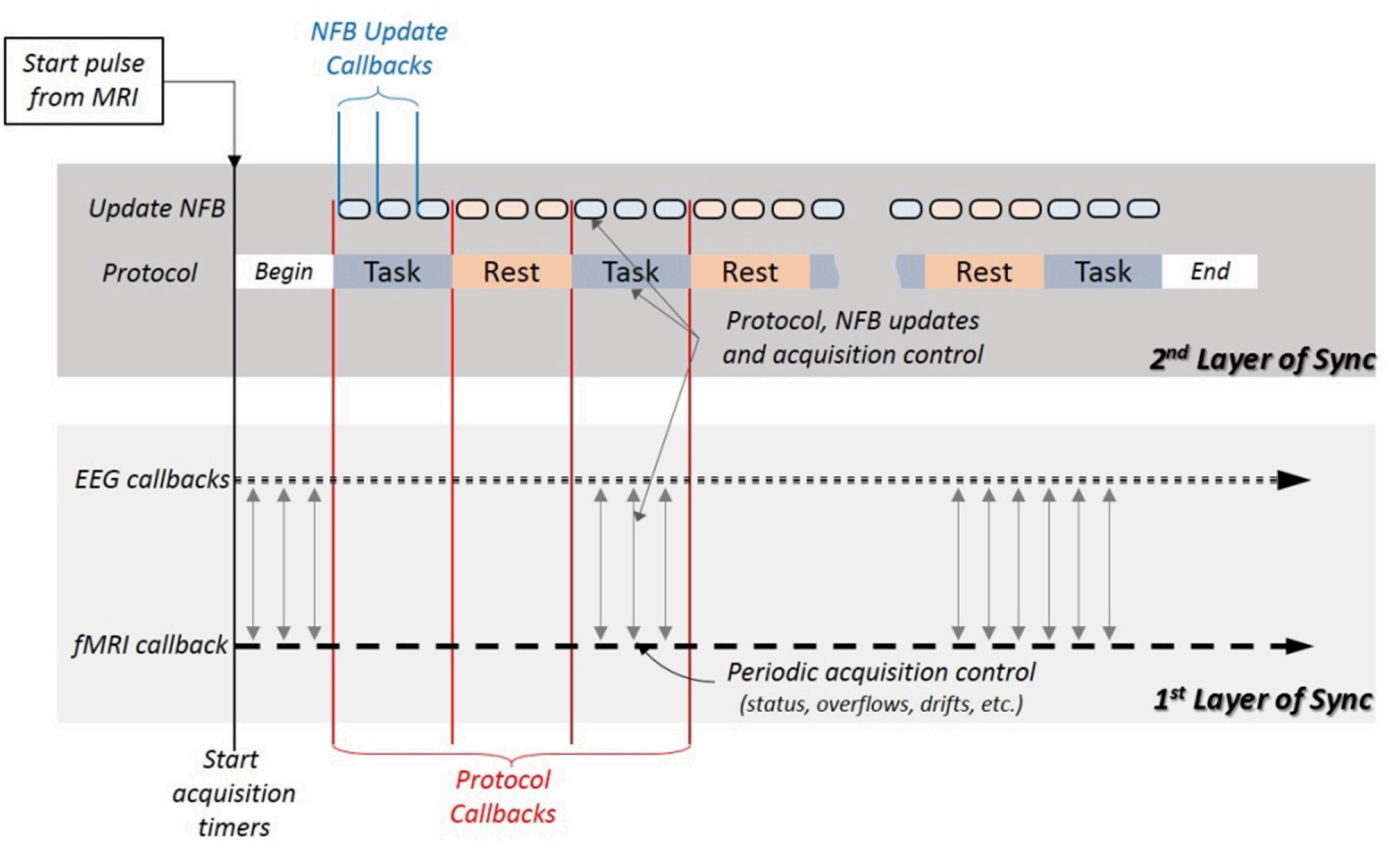
**The system synchronization is divided into two layers**. The first layer synchronizes the acquisition subsystems by using EEG and fMRI callbacks, and issues periodical controls for de-synchronization, all independently from the NFB protocol. The second layer relies on the synchronization of the first layer, and uses protocol and update NFB callbacks to ensure the synchronization of the protocol with the acquisition subsystems, the NFB calculation and visualization. It uses protocol, update and synchronization controls to detect de-synchronization.

In bimodal NFB, the simultaneous signals coming from each modality should reflect the brain activity occurring during the specific task indicated by the protocol with minimal delay or drift. This demands a high level of synchronization between both subsystems and the protocol. One strategy to achieve such synchronization is by dividing it into two different layers. The first layer shall be responsible for the acquisition subsystems and the second one for the protocol (see Figure 5).

##### 2.1.4.1. Acquisition synchronization (first layer)

The synchronization starts with the acquisition of the first fMRI scan. At the beginning of each acquisition, the MR platform sends a TTL pulse which marks the start of the online session and is registered as the time reference for all the following events. Immediately after the first pulse, the *NFB Unit* starts collecting data from both subsystems using their respective callbacks. To provide real-time acquisition with virtually no delay, the callbacks' frequencies should be equal or higher than their respective subsystems' acquisition frequencies. For example, if EEG is digitized at 250 Hz then the callbacks should be ≥250 Hz. Similarly, for fMRI acquisitions done at 1Hz the callbacks should be ≥1Hz. For practical reasons both callbacks can be set to the highest acquisition frequency (i.e., EEG's). When the NFB protocol is not highly time sensitive, the callback frequencies can be set lower than that of the EEG sub-system in order to allocate more computing time and power for data processing.

In this layer, both the EEG and the fMRI signals have the same starting time marked by the first TTL and and are collected synchronously. To ensure continuous synchronization, periodic checks should be implemented in the *NFB Unit*, such as buffer overflow, acquisition delays or drifts.

##### 2.1.4.2. Protocol synchronization (second layer)

This layer is necessary for the synchronization of the NFB calculation with the acquisition subsystems, and to guarantee that the NFB shown to the subject corresponds to the brain activity that was measured following the experiment protocol.

In general, a NFB experiment requires the subject to perform a specific mental task that changes the targeted brain activity. An example protocol would be to repeat a specific task several times and separate those repetitions with some rest period where the subject can reduce his/her mental activity. Depending on the protocol, at the beginning of the experiment the task interval duration can be either fixed or variable, whilst the rest duration is usually fixed. Thus, the above protocol might be implemented in the two following ways:

- *Fixed task interval*. The task duration is fixed and well known before the experiment starts. For example, a task interval of 20 s duration is followed by a rest interval of 20 s duration and this block is repeated 10–15 times throughout the session.
- *Flexible task interval*. The task duration is variable (i.e., *task*∈*[5,60] s*) with respect to the NFB result. This means that the task will continue until a certain NFB target is achieved. Only when this target is achieved there will be a shift into a rest interval of known duration (i.e., *rest*∈*[5,40] s*). This procedure can be repeated 10–15 times throughout the session.

The NFB value presented to the subject is updated periodically (i.e., every 500 ms) by the Update NFB callbacks. To ensure synchronization with both acquisitions, these callbacks start simultaneously with the EEG and fMRI callbacks, right after the first TTL pulse.

Switching between task and rest intervals is controlled by the protocol callbacks. To ensure synchronization, the protocol callbacks should be triggered simultaneously with their corresponding Update callback, at the end of each interval. This requires for the protocol callback period to be a multiple of the Update NFB callback period. In the fixed task interval example above the protocol callback period is invariable (20 s), thus the Update NFB can be easily set at 500 or 200 ms. In contrast, the second example has a variable protocol callback period. In this case, for synchronization purposes, the protocol period duration will be set at the end of the Update NFB callback that occurs right after the target is achieved.

At this point, the whole system is synchronized from the acquisition to the protocol. Periodic controls in each layer and between layers should be implemented to ensure synchronization throughout the session. If abnormalities occur, they should be reported and when possible dealt with.

### 2.2. Neurofeedback presentation

The choice of communication device depends on the type of NFB that is being used. Screens or goggles are used for visual, headphones for aural and tactile devices for tactual NFB presentations. All these devices must be MR compatible. In this paper we focus on visual presentations, but the same principles can be transferred to any type of presentation and sensing modalities.

**Figure 6:**
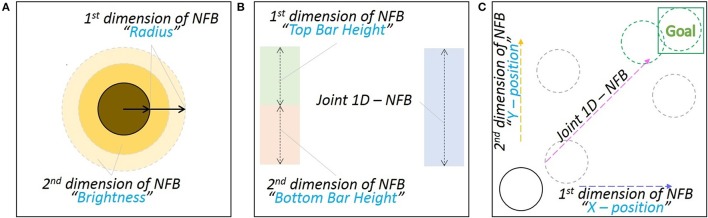
**Neurofeedback visualization examples: (A)** Sun metaphor; the Sun's brightness is controlled from one NFB value (EEG-NFB) whilst the radius from the other value (fMRI-NFB). Both the brightness and the radius can be proportionally changed when only one NFB value is used. **(B)** Two variation of bar representations; the addition of the normalized NFB values or any of them independently can control the height of the bar. **(C)** The NFB values control the *x* and *y* positions of the disk. When only one NFB value is used, both coordinates change proportionally.

The NFB presentation needs careful consideration. Complex visualizations might not help the subject or even interfere with the mental task that is being performed during the experiment, instead, simplicity is commonly preferred. Depending on the NFB dimensions, there can be various visualization approaches. For example, when a two dimensional visualization is needed, a “Sun” metaphor (see Figure 6A) can be used, where the diameter changes based on one of the values (i.e., fMRI) and the brightness changes based on the other (i.e., EEG). The bars in Figure 6B can be used to represent either one (left) or two (right) dimensional NFB. The animation that uses the motion of the circle into the goal rectangle (see Figure 6C) is another two dimensional example. Furthermore, all these representations can be adapted for one dimensional scenarios, by keeping one visualization feature constant and changing the other or simultaneously change both features proportionally to the NFB value.

Finally, with the display, the NFB loop introduced in Figure 1 is now closed; the NFB is sent back to the patient and the patient is able to change its brain activity based on the received NFB. Selecting and integrating together all the components introduced throughout this section is a challenging technical task. So far we have described the different parts of a bimodal platform and their functionality. In the following section we are going to introduce the hybrid EEG and fMRI platform that we have built, which is currently being used for bimodal NFB experiments.

## 3. Illustrative example: bimodal neurofeedback platform at neurinfo

**Figure 7:**
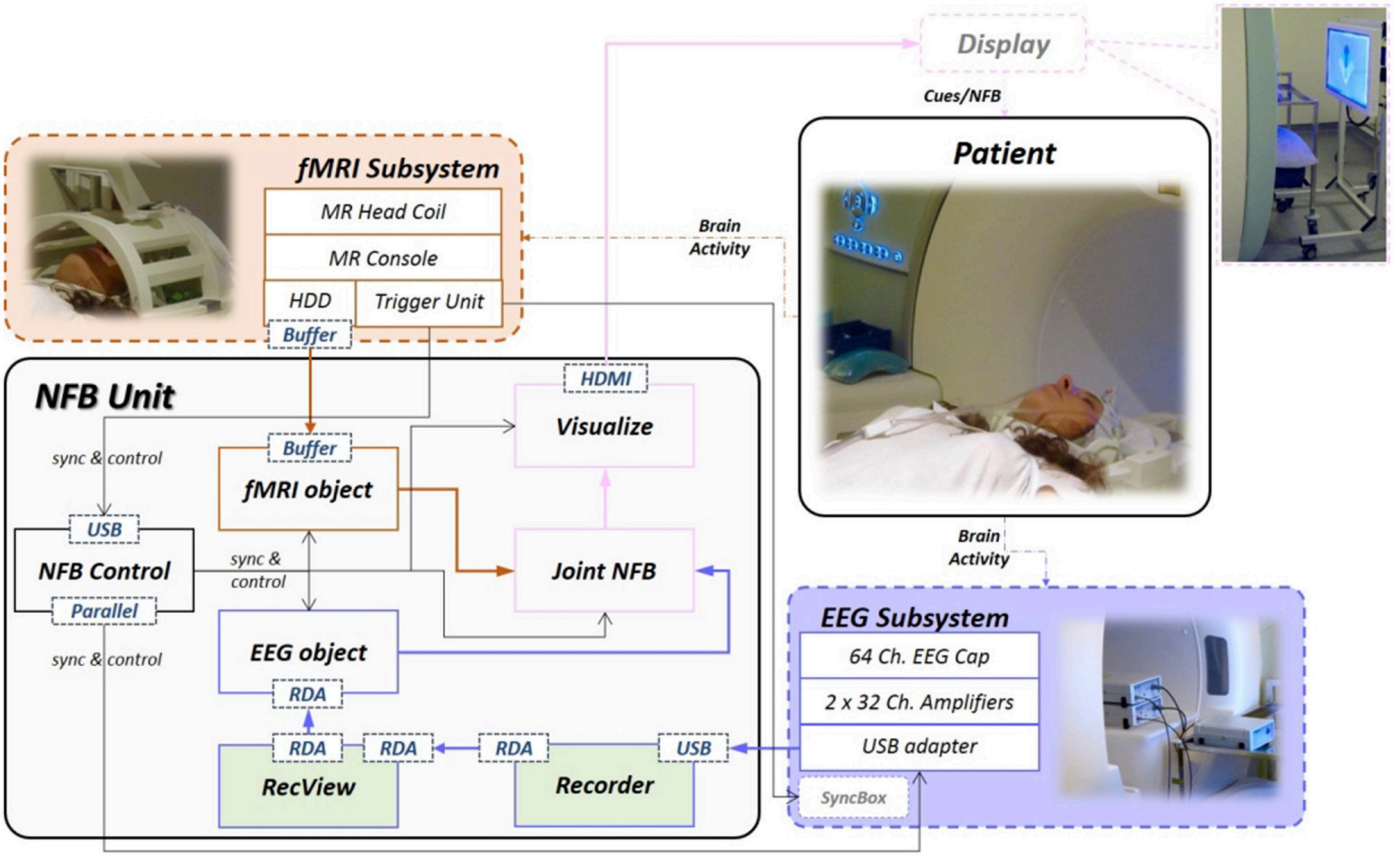
**The detailed diagram of the hybrid EEG-fMRI bimodal NFB platform at Neurinfo**. The EEG (in purple) and fMRI (in orange) signal flow includes the respective subsystems and software modules inside the *NFB Unit*. Both pipelines merge at *Joint NFB*, which calculates NFB and then sends the results to *Visualize*. *Recorder* and *Recview* are the only commercial software, the rest of the *NFB Unit* modules are developed in-house (Matlab/C/C++/Java). The *NFB Control* exchanges synchronization and control information with the rest of the hardware and software components.

In this section we are going to describe the hybrid EEG-fMRI NFB platform that we have developed and used in our NFB experiments (see Figure 7).

### 3.1. EEG subsystem

**Figure 8:**
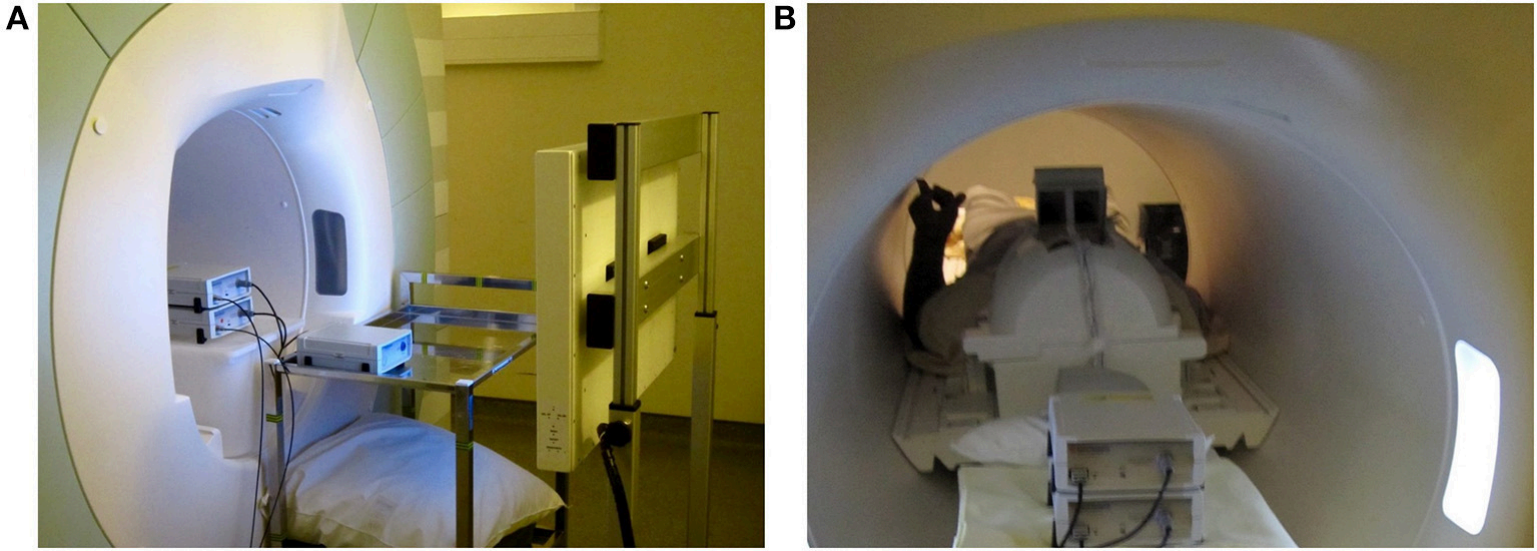
**System installation pictures. (A)** Placement of amplifiers, battery and LCD display. **(B)** Placement of the rear view mirror on the top of the head coil.

Our EEG subsystem is an MR compatible solution from Brain Products^3^. The EEG signals are acquired with a 64-channel cap, equipped with a drop-down electrocardiogram electrode for heart pulse measurements. The cap is connected with two 32-channel battery powered amplifiers via two electrical cables. During experiments, the battery and the amplifiers are placed inside the bore right behind the subject's head (see Figure 8). The amplifiers use fiber optic cables to send the digitized signal to a USB adapter and then to the *NFB Unit*. The USB adapter is also connected with the 10 MHz clock of the MR scanner's gradient switching system, via the SyncBox. This connection is necessary for the phase synchronization needed for the MR artifact correction (see Section 2.1.1). Furthermore, the *NFB Unit* communicates with the USB adapter via parallel connection. The parallel connection is used to send triggers that timely mark the EEG data, for online synchronization control and for offline data analysis. It is worth noting that the installation of the EEG system is done according to the manufacturer recommendation and that different manufacturers might provide different guidelines.

### 3.2. fMRI subsystem

Our fMRI subsystem is a Nordic-Neurolab (NNL) solution with a Siemens 3T MR scanner. The MR imaging is performed on a Siemens MR scanner (Magnetom 3T Verio, Siemens Healthineers, Erlangen, Germany, VB17) with a 12-ch head coil allowing secure installation of the EEG cap and connection of the bundle to the amplifiers. The NNL hardware solution is used for visual stimulation and synchronization between the MR console and the *NFB Unit*. Furthermore, our platform relies on its *Trigger Unit* for the TTL trigger that is sent during MR acquisition.

### 3.3. *NFB Unit*

All the software modules of the *NFB Unit* are deployed on two PCs connected on the same LAN with the MR console. Generally, brain activity measurement systems have their corresponding commercial acquisition software. The usage of manufacturer's software in most cases is not only obligatory but also a convenient way to achieve optimal real-time signal acquisition.

#### 3.3.1. Acquiring real-time EEG data into the *NFB Unit*

In our experiments, *Recorder* is used before the experimental session to configure and setup the EEG acquisition (i.e., channel montages, impedance measurement). Then during the experiments it receives data from the USB adapter (see Section 3.1), pre-filters it and then forwards it for further real-time processing to *RecView* (or similar platforms like Matlab^4^ or OpenViBE^5^) using the built-in TCP/IP based Remote Data Access (RDA) feature. Simultaneously, it saves the raw EEG data, the acquisition parameters and the setup information.

*RecView* has specific filters to remove the gradient and the BCG artifacts from the EEG signals and an additional RDA interface to transfer the data to other EEG processing software. The *NFB Unit*, collects the data using Matlab (i.e., the *EEG object*), but we have also successfully tested the interface to send real-time EEG data to OpenViBE. The *EEG object* uses the TCP/UDP/IP Matlab Toolbox (pnet^6^) to communicate with *RecView*. The communication protocol is straightforward. At the beginning the RDA server sends the header with the “START” message and the setup information (i.e., number of channels, channel labels, sampling interval). Then, it continuously sends the EEG signals with their event markers, and finally the “STOP” message when the acquisition is stopped.

#### 3.3.2. Acquiring real-time fMRI data into the *NFB Unit*

The fMRI acquisition is done by certified MR technicians using the MR console software (see Section 3.2). Few sequences are used for imaging depending on the experimental protocols and EEG-fMRI acquisition safety guidelines. All the fMRI series are stored in the console's hard drive at the end of each acquisition.

To the best of our knowledge there does not exist a universal way to acquire real-time fMRI data from all types of scanners, thus it is highly recommended to contact directly the scanner vendor for any available options and/or configurations that could be used. Two ways for real-time fMRI acquisition that were investigated and tested with our Siemens system, are described here.

- The scanner's software can be configured (using “*ideacmdtool*”) to sequentially export single fMRI scans in “dicom” format at a predefined folder using FTP protocol. Then an acquisition software can monitor for new files (i.e., using *FileSystemWatcher*^7^ library). In our observations we have noticed jitter in file export, which was more significant for sequences with TR below 2 s.
- During an fMRI acquisition, each newly acquired volume's raw data is saved in the console's hard drive. To retrieve these raw data we use a TCP/IP buffer solution from FieldTrip^8^. In brief, this solution consists of an executable server, deployed into the scanner host, and a client running on the *NFB Unit*. The server reads each new file and sends it to the client buffer that can be accessed from Matlab.

In our platform, the later method is employed to transfer the fMRI data over TCP/IP into the *NFB Unit* (i.e., the *fMRI object*).

#### 3.3.3. Processing the EEG and the fMRI data

The EEG signal processing is handled by the *EEG object*. This object is created at the beginning of each experiment and contains all the necessary members to store the signals, events, setup information together with the initialization information and updates. Furthermore, it has additional methods that perform various signal pre-processing, spatial and temporal filtering, and feature extraction (see Section 2.1.1). Similarly, the fMRI data is handled by the *fMRI object*.

In both objects, the extracted features are assigned to respective object's public members in order to be accessible by the *Joint NFB*. The *Joint NFB* contains calculation methods (i.e., percent signal change, z-Score) for either unimodal or bimodal scenarios. Furthermore, it is equipped with various configuration variables that simplify the optimization of the existing models and templates for the implementation of new ones.

The estimated NFB values are used by *Visualize* (see Figure 7), which controls the display that communicates with the subject. *Visualize* has a collection of visual objects, developed in Psychtoolbox^9^, for: explaining the NFB tasks (i.e., texts), showing cues and for animating the NFB representation (i.e., 2D/3D objects). It also contains additional audio and visual objects used to communicate with the subject throughout the experiment for various instructions and notifications.

#### 3.3.4. Control and synchronization

The last part of the system, the *NFB Control*, is a class object developed in Matlab and Java^10^. This object is responsible for starting/stopping the experiment, controlling all other objects' behavior throughout the experiment, synchronization, and finally saving all the experiment data.

The *NFB Control* constructor is initialized with protocol information (i.e., tasks or conditions, duration, repetition). The input can be given through a GUI for few standard protocols or with custom scripts for more specific ones. The *NFB Control* initializes all the objects necessary for the experiment based on the requirements of the input protocol. Thus for unimodal NFB only one of the *EEG* and *fMRI objects* will be initialized, whereas for bimodal NFB an *EEG objects* and an *fMRI objects* will be initialized with their respective initialization information. Furthermore, it also defines the method used in *Joint NFB* and initializes the *Visualize* objects that are going to be used for presentation.

*NFB Control* receives synchronization information from both subsystems, from the *Trigger Unit* of the fMRI subsystem and through *RecView* from the USB adaptor of the EEG subsystem (see Figure 7). At each fMRI volume acquisition the scanner sends a TTL signal from the *Trigger Unit*. When *NFB Control* receives the first TTL signal, it starts the acquisition callback function(s) (see Figure 5). After the “Begin” period which is predefined by the protocol, it starts the rest of the callback functions and when the session is over, it stops all the callback functions and saves the data.

The EEG subsystem records scanner's TTL signals to correct the MR artifact in *RecView*, thus the EEG data coming from *RecView* already contains the fMRI volume markers. Furthermore, the *NFB Control* uses a parallel connection to the USB adapter to send markers to the EEG signals at each protocol callback. These protocol markers are then resent together with the rest of the EEG data to the *EEG object*, with a pre-measured delay that in our implementation is 38–40 ms.

All the EEG markers including protocol markers and TTL pulses coming from the scanner are used to periodically control for delays in both layers of synchronization (see Section 2.1.4). The TTL markers are used to check for fMRI acquisition delays or jitter. The same markers, which are recorded on the EEG data for MR correction (see Section 3.1), are used to check for delays in the EEG acquisition and that both subsystems are acquiring data synchronously. The protocol callback markers are used to control the synchronization of the NFB updates, and to make sure that the data that is used for the NFB update was acquired while the subject was performing the task required by the protocol. When a de-synchronization occurs, the NFB Control reports it and tries to re-synchronize. If the re-synchronization attempt is unsuccessful the current session is stopped and the stack data is saved.

### 3.4. Display

The communication with the subject lying on the back in the MR bore, is done via an LCD Screen and a rear-facing mirror fixed on the top of the head coil (see Figures 8A,B). The 32-inch LCD screen is part of the NNL solution (see Section 3.2); it has a 60 Hz input refresh rate and is connected with the NFB Unit via fiber optic using a DVI to fiber optic converter and powered by an MR compatible power supply.

### 3.5. Real-time performance

**Figure 9:**
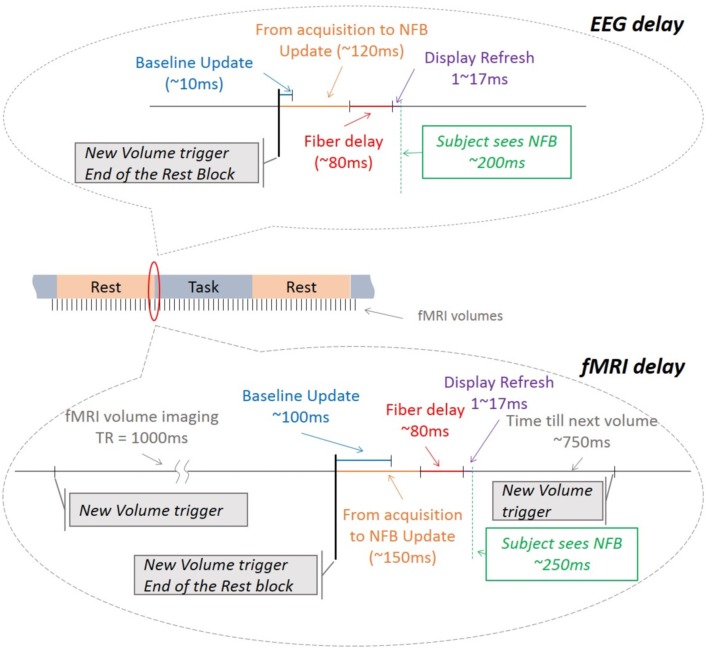
**Timeline description of all the hardware and software delays for EEG and fMRI**. The values include the manufacturer's descriptions and/or the results of the measurements performed in our lab.

Real-time tests and experiments have shown very good performance with various pre-processing, filtering, NFB calculation and visualization methods. The entire fMRI process from acquisition to NFB update takes ≃150 ms, well below the TR of regular EPI sequences (see Figure 9). The NFB visualization is very fast (1–2 ms) and it is done within one screen refresh (i.e., 16, 7 ms for a 60 Hz screen). The screen inside the scanner is connected to the *NFB Unit* via optic fiber which minimizes the delay at 80 ms (according to the manufacturer's recommendation). Thus the fMRI NFB is shown to the subject with a total delay of ≃250 ms. For EEG this delay is ≃200 ms.

Furthermore, at the end of every rest interval the baseline is updated in both EEG and fMRI. These updates take on average less than 20 ms for EEG and less than 100 ms for fMRI, which is lower than their respective NFB update cycles. These delays do not affect each other because: (1) the model updates are done in parallel with the processing of the respective signals and, (2) the EEG and the fMRI pipelines work in parallel.

## 4. Setup and neurofeedback experiments

The hybrid EEG and fMRI platform has been already successfully used to conduct several unimodal and bimodal NFB studies. The goal of the used NFB protocols has been to maximize the brain activity measured by EEG and fMRI while performing Motor Imagery (MI) tasks. This section briefly describes all the steps followed to prepare and then to perform bimodal NFB experiment with our platform.

### 4.1. Preparation

**Figure 10:**
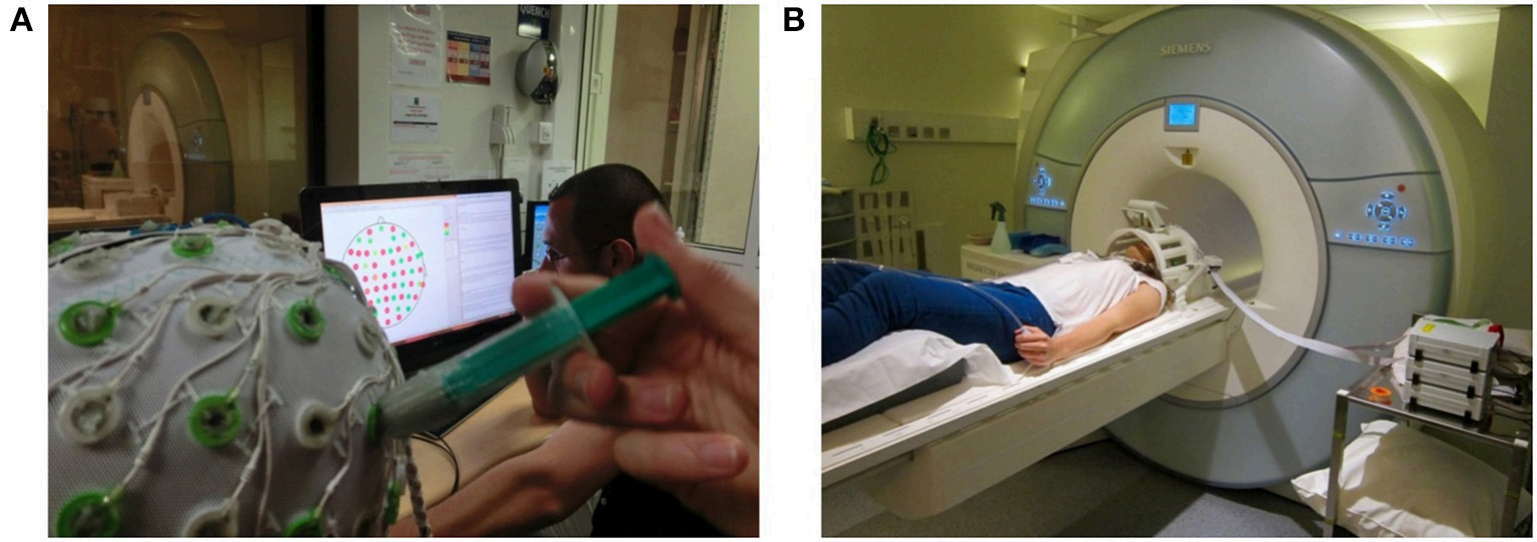
**Subject's preparation and subsystem installation before the experiment. (A)** EEG subsystem installation and impedance check outside the MR room, **(B)** installation of the MR coil and EEG impedance recheck.

At the beginning of each experiment, outside the MR room, a 64 channel EEG cap with adequate size was fitted on the subject's head and conductive gel was applied until electrode impedances were below 10 kΩ. Next, the recording configuration was set up and tested until the acquisition was working properly. Then, the system was disconnected and placed inside the scanner room (see Figure 10). Meanwhile the subject was also put inside the scanner. At this stage, a secondary test was done to control whether the acquisition was still working and that electrodes' impedances had not changed due to the subjects' movements. This procedure was repeated until the acquisition was working, the impedances were within range, and the subject was ready for MRI scanning.

### 4.2. Structural scan

A high resolution structural 3D T1 was acquired with an MPRAGE sequence with TR/TI/TE = 1,900/900/2.26 ms, GRAPPA 2, 256 × 256 mm^2^ Field of View and 176 slabs, 1 × 1 × 1 mm^3^ voxel size. This scan was later used for offline analysis.

### 4.3. Initialization

The NFB initialization (see Sections 2.1.1 and 2.1.2) was done on a non-NFB session with block-design task with 20 s alternate periods of “Rest” (the subject is asked to try to minimize brain activity) and “Task” (the subject is asked to perform motor imagery) that was performed prior to the online NFB sessions.

### 4.4. Non-NFB session

This is basically a calibration session for NFB. The task-related EPI acquisitions were taken with TR/TE = 2,000/23 ms, 210 × 210 mm^2^ field of view, 2 × 2 × 4 mm^3^ voxel size and 16 (or 32) slices. This fMRI series underwent a GLM analysis to extract the activation maps and then to localize a brain ROI with the highest protocol related activity. Such GLM analysis can be performed by the MR software (Syngo MR) by pre-specifying the paradigm on the console or externally on a separate computer (i.e., using SPM, FSL ^11^or AFNI ^12^). The later method usually gives more accurate results but it is far more time consuming; hence, a trade-off shall be made between time and accuracy when the subjects are inside the scanner. Furthermore, an initial baseline as a relative change to the BOLD contrast likelihood inside the ROI was established based on the statistical analysis of the calibration data of all the “Rest” intervals' volumes. To account for global changes in the brain activity, a background region (i.e., the upper slice of the fMRI volume) is also taken into consideration. Its baseline was also calculated offline.

Simultaneously, the EEG signals were recorded during the calibration session. After artifact removal and filtering the data was segmented into 20 s “Task” and “Rest” epochs. Then all epochs were re-segmented into 2 s windows with 95% overlap and used to train spatial filters via CSP algorithm. Similar to fMRI, an initial NFB baseline target was calculated by averaging the EEG features extracted with CSP filters from all the rest intervals. This baseline was used at the very beginning of the NFB sessions and then updated later online after each 20 s “Rest” period.

### 4.5. NFB sessions

**Figure 11:**
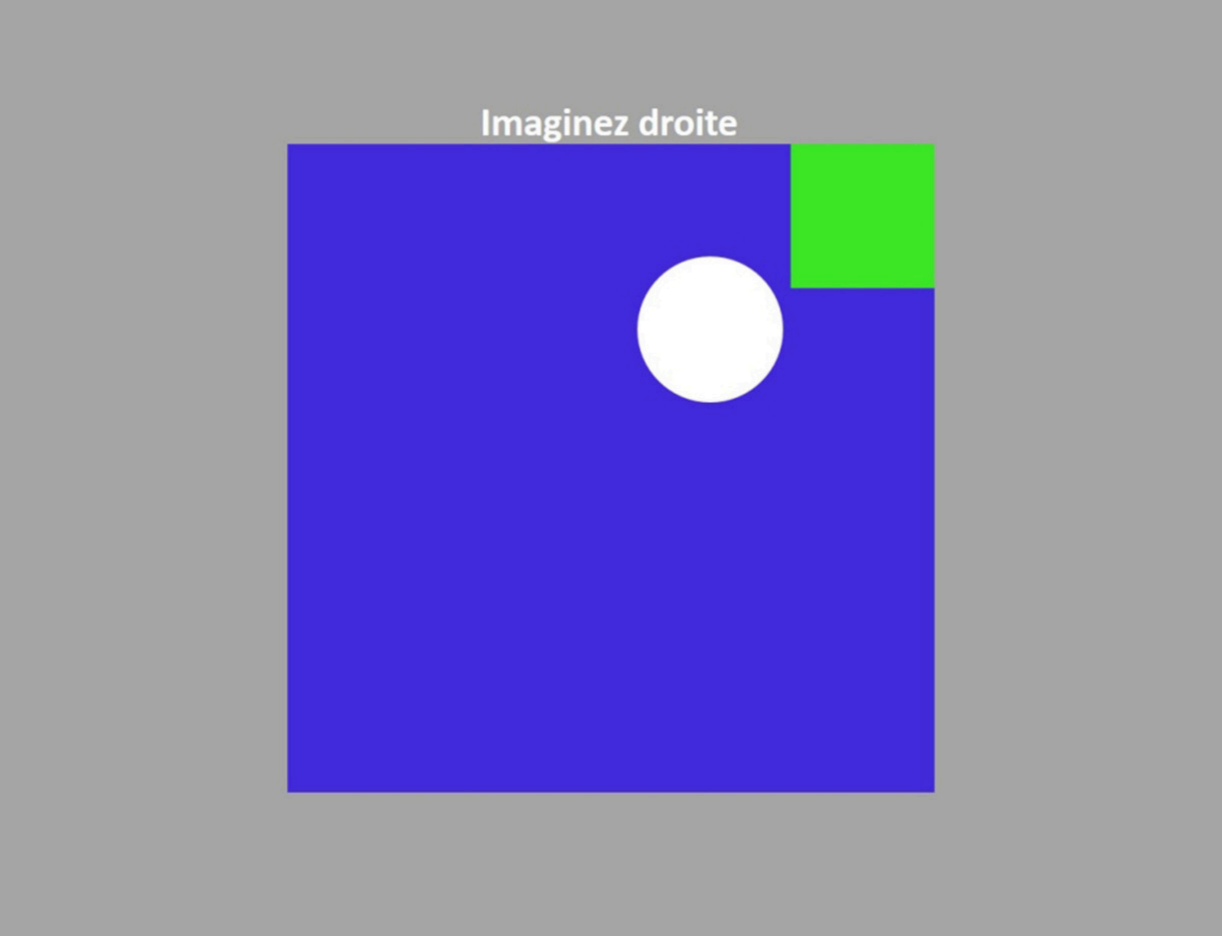
**Snapshot of the subject's display during a bimodal NFB session**. The brain activity measured with EEG and fMRI is represented by the white disk movement inside the blue square. The goal is to position the disk over the little green square, which in this case is achieved when the normalized NFB values coming from both EEG and fMRI are simultaneously maximized with respect to their NFB targets.

We have successfully used our platform to conduct two experimental studies with more than 100 NFB sessions and more than 30 subjects. Preliminary experimental results are shown by Perronnet et al. (2016). In the first study we compare the usage of bimodal NFB vs. unimodal NFB for self-regulation of brain activity of 8 subjects. The second study aims to quantify the ability to improve self-regulation of the brain activity when performing repeated bimodal NFB sessions in 24 healthy subjects. Overall, the performance of the platform has been very satisfactory and the experiments have shown that the subjects were able to regulate their brain activity using visual NFB (see Figure 11).

The fMRI-NFB was calculated for every new volume. For pre-processing we have used spatial smoothing, motion correction and slice-time correction. The initialization ROI was used to spatially filter the fMRI volumes and the baselines were used to estimate the average percent signal change (APSC).

The EEG-NFB was calculated every 250 ms over the most recent 2 s window. The gradient and BCG artifacts were removed with online updated templates. Then the signals were spatially filtered with CSP and band pass filtered in [8, 30] Hz. The band power estimation was used to compute the NFB value using APSC. During the NFB session all the baseline values for both EEG and fMRI were updated at the end of each “Rest” block.

To conclude, the experimental studies conducted with our platform have shown that it is possible to build and use such hybrid systems for bimodal NFB experiments. The technical difficulties associated with the field can be addressed and it is possible in the future to perform experiments with various unimodal or bimodal NFB protocols for both research and clinical studies.

## 5. Discussion

Multimodal brain activity monitoring has the potential to not only improve NFB but to also provide a better understanding of the brain functionality. But, the simultaneous acquisition and processing of two or more types of neurosignals in real-time can be very challenging. From a technological and safety point of view the challenges of the simultaneous EEG and fMRI acquisition have been addressed before (Ullsperger and Debener, 2010; Neuner et al., 2014; Bannier et al., 2015), instead the focus of this paper is on the utilization of the existing EEG and fMRI subsystems in order to build a platform that is capable to perform real-time bimodal NFB experiments.

The design and implementation of a hybrid EEG and fMRI platform that is capable of acquiring signals, processing, modeling, estimating NFB and then communicating with the subjects in real-time has to be carefully considered. Two very different hardware and software subsystems need to work together, fully synchronized and without compromising real-time performance. Throughout the paper we have particularly emphasized the need for real-time performance and synchronization. We have chosen a two-layer synchronization approach in order to simplify the implementation and to allow flexibility to use the platform for both unimodal and bimodal NFB protocols.

Our platform relies on network communication and its modular architecture offers the possibility of distributing the system on different processing units. Inevitably, slow network connection or network congestion might introduce delays in data transmission and for highly time sensitive protocols networking and data transmission aspects need careful consideration. In our implementation, the networking delays, which include the signal acquisition and processing delays (see Figure 9), rely mainly on the manufacturers' guidelines. Few non-exhaustive tests that we have conducted in general confirmed the manufacturers' claims.

A similar platform for bimodal EEG and fMRI NFB was reported by Zotev et al. (2014). Beyond the choice of subsystem' manufacturers, operating environment and custom software packages, their platform architecture, components and functionality with respect to the NFB process flow, are very similar to the platform introduced here. Their real-time processing pipelines use also similar methods to calculate the respective NFB values. The NFB is also presented visually, but in contrast with our platform is shown separately for each modality.

A very important future goal in the field of bimodal brain activity monitoring and multimodal NFB is the development of good coupling models. Such models have the potential to maximize the information that is extracted from each modality and put it in the context of better understanding the underlying physiological brain activities. This can be particularly advantageous in the study of neuronal source localization from the EEG inverse problem, which recently is being combined with fMRI data in a bimodal framework. Its main applications are the localization of epileptic foci, the analysis of sources in selected frequency bands in psychology, or any other application where a precise spatio-temporal detection can be the point of interest. A major limitation for these studies has been the experimental setup aspects of simultaneous EEG and fMRI recording. Due to these difficulties, some researchers have opted to record the data in separate sessions, or use experimental techniques which lead to data with very low signal-to-noise ratio. Different applications develop models for EEG and fMRI integration that are only evaluated on the synthetic data due to the lack of real synchronous data. Our platform addresses the above problems by providing fully synchronized simultaneous acquisition and by offering easy integration of both modalities at all processing stages. For this reason, the EEG and fMRI objects provide public members to store the results of each processing step that can be accessed and used in future implementations of coupling models. Furthermore, the initialization information can be customized to input specific modeling information that might be needed for future developments.

There exist also applications that might require additional non neural bio-signals that indirectly represent an estimation of brain activity. For example the galvanic skin response can be used to monitor the stress level of a NFB subject. Furthermore, auxiliary sensors can be used to provide additional information for NFB or even for monitoring other aspects of the experiments. For example electromyography can be used to monitor the subject's muscular activity when and if a NFB protocol requires it. Motion cameras or sensors can be used to better measure the subject's head motion, which currently is estimated by a least squares approach based on the 6 parameter (rigid body) spatial transformation (Friston et al., 1995).

The addition of any new real-time signals needs to be carefully considered in terms of synchronization and computing power. In the current state of the platform the synchronization is solved by using two hierarchical layers. A major advantage of this approach is the possibility to synchronize additional signals with minimum effort, by using the existing layers' infrastructure for acquisition and protocol synchronization. On the other hand, the additional computational power need to be estimated carefully before choosing the hardware/software configuration. In our implementation, the real-time fMRI processing is the most computationally demanding. With the current hardware configuration there are limitations in the analysis that can be performed in real-time. In the near future, we intend to use a GPU cluster and take advantage of its parallel processing power to perform standard GLM and ICA analysis on full volume fMRI series, or even recent more advanced local multivariate detection methods such as a contrario (Maumet et al., 2016), in real-time.

As we showed in this section, there is still remaining challenges and difficulties for improving real-time multimodal brain activity measurement but although not yet very common, the increasing research interest will provide a wide range of applications for multimodal brain research, and many more similar or even more capable platforms should emerge in the following years.

## 6. Conclusion

We described a general method for setting up a hybrid EEG and fMRI platform for bimodal NFB experiments. Our scope is to help researchers build faster and robust platforms, and also provide some minimal technical requirements or features to look for in future commercial systems. Based on guidelines described here, we have implemented a hybrid EEG and fMRI bimodal platform. So far, our platform has been successfully used in two experimental trials with more than 100 NFB sessions and more than 30 subjects.

## Author contributions

MM, AL, EB, LP, and CB prepared the technical requirements of the platform. MM designed, developed and implemented the platform. MM and EB investigated and then developed the real-time fMRI acquisition. MM and LP designed the NFB protocols and the NFB calculation. MM wrote the majority of the manuscript. MM and SN co-wrote Sections 1 and 5. MM and EB co-wrote Section 3.2. AL and CB supervised all the aspects of the research and the platform development, and reviewed in depth the manuscript. All the authors read and approved the final manuscript.

## Funding

The MRI data subsystem is part of the the Neurinfo MRI research facility from the University of Rennes I. Neurinfo is granted by the European Union (FEDER), the French State, the Brittany Council, Rennes. The EEG subsystem and the bimodal platform development has received French government support granted to the CominLabs excellence laboratory and managed by the National Research Agency in the “Investing for the Future” program under reference ANR-10-LABX-07-01. It was also financed by Brittany region under HEMISFER project.

### Conflict of interest statement

The authors declare that the research was conducted in the absence of any commercial or financial relationships that could be construed as a potential conflict of interest.

## Footnotes

^1^http://www.neurinfo.org/.

^2^http://www.fil.ion.ucl.ac.uk/spm/.

^3^http://www.brainproducts.com/.

^4^https://fr.mathworks.com/products/matlab/.

^5^http://openvibe.inria.fr/.

^6^http://www.mathworks.com/matlabcentral/fileexchange/345-tcp-udp-ip-toolbox-2-0-6.

^7^https://msdn.microsoft.com/en-us/library/system.io.filesystemwatcher(v=vs.110).aspx.

^8^http://www.fieldtriptoolbox.org/development/realtime/fmri.

^9^http://psychtoolbox.org/.

^10^http://www.java.com/.

^11^https://fsl.fmrib.ox.ac.uk/fsl/fslwiki.

^12^https://afni.nimh.nih.gov/afni/.
